# Independent, Controllable Stretch-Perfusion Bioreactor Chambers to Functionalize Cell-Seeded Decellularized Tendons

**DOI:** 10.1007/s10439-019-02257-6

**Published:** 2019-04-08

**Authors:** Giuseppe Talò, Daniele D’Arrigo, Sergio Lorenzi, Matteo Moretti, Arianna B. Lovati

**Affiliations:** 1 Cell and Tissue Engineering Laboratory, IRCCS Istituto Ortopedico Galeazzi, Via Riccardo Galeazzi 4, 20161 Milan, Italy; 2 Department of Engineering and Applied Sciences (DISA), University of Bergamo, Dalmine, Italy; 3 Regenerative Medicine Technologies Lab, Ente Ospedaliero Cantonale, Lugano, Switzerland; 4 Cardiocentro Ticino, Lugano, Switzerland

**Keywords:** Uniaxial bioreactor, Tissue engineering, Dynamic culture, Collagen matrix

## Abstract

Tissue-engineered decellularized matrices can progress clinical replacement of full-thickness ruptures or tendon defects. This study develops and validates a custom-made automated bioreactor, called oscillating stretch-perfusion bioreactor (OSPB), consisting of multiple, independent culture chambers able to combine a bidirectional perfusion with a programmable, uniaxial strain to functionalize cell-seeded decellularized tendons. Decellularized tendon matrices were seeded on their surfaces and within the tendon fibers with mesenchymal stem cells. Then, they were subjected to a bidirectional perfusion and programmed stretching cycles of 15–30–60 min on–off two times per day for 7 days of culture. *In vitro* analyses showed viable cells, homogenously distributed on the surface of the constructs. More importantly, cell-seeded decellularized tendon grafts undergoing cyclic load in our bioreactor had a superior production and organization of newly formed collagen matrix compared to static cultured constructs. The coherency and local alignment of the new collagen deposition within the inner injected channels quantitatively supported histological findings. The designed OSPB could be considered a unique, cost-effective system able to involve multiple independently controlled chambers in terms of biological and mechanical protocols. This system allows parallel processing of several customized tendon constructs to be used as grafts to enhance the surgical repair of large tendon defects.

**Electronic supplementary material:**

The online version of this article (10.1007/s10439-019-02257-6) contains supplementary material, which is available to authorized users.

## Introduction

Tendon injuries and full-thickness ruptures represent common occurrences in musculoskeletal disorders, affecting patients’ quality of life and treatment costs. With a low metabolic activity, tendons have a sub-optimal capability to heal with enhanced risks of re-injuries. In these cases, graft replacement is the gold standard approach. Synthetic and natural biomaterials have been investigated as tendon substitutes. Among several types of grafts, research is focused on decellularized matrices derived from humans or animals to better resemble complex biochemical, ultrastructural and mechanical properties of native tendons.^5^ A good removal of cell-related immunogenicity of these grafts can be obtained through decellularization protocols.^2^,^8^,^29^ However, these biological, non-vital implants are prone to degeneration and limited duration after implant.^11^ Therefore, decellularized matrices should be reseeded with autologous cells to improve viability and long-term duration, as well as mechanical properties and host integration of implanted grafts.^25^ The combination of decellularized biological matrices reseeded with host cells can guarantees the cell migration and distribution while preserving the physiological architecture and alignment of collagen fibers.^25^ Nevertheless, the highly dense collagen organization of decellularized tendon matrices hinders the cellular repopulation throughout their 3D structure.^5^

To overcome this limitation and to produce functionalized tissues *in vitro*, functional tissue engineering (FTE) combines autologous cells and biological scaffolds with proper biochemical and physical stimuli using bioreactors.^9^ Bioreactors are dynamic culture systems able to strictly control the local microenvironment by providing nutrients for cell metabolism and turnover, and delivering mechanical stimuli that regulate the construct homeostasis *in vitro*.^25^ The use of bioreactors better supports cell alignment and differentiation as well as the extracellular matrix (ECM) deposition and organization along decellularized grafts.^9^,^13^,^25^ However, in FTE, the high variability of mechanical stimuli, culture substrates and cell sources impedes the protocol standardization.^13^ In addition, the heterogeneity of structural and biomechanical results makes difficult to compare studies in this field. Hence, for a successful translational approach, independent, programmable and automatic dynamic systems can represent a step forward in FTE permitting to individually control each sample and each condition.

Several authors describe the use of dynamic systems to culture decellularized tendon matrices after cell reseeding.^8^ Most of them employed rotating tube mixers in the absence of mechanical stimuli to culture different cell types onto decellularized tendons.^7^,^15^,^20^,^27^ However, a suitable mechanical stimulation is required to induce cell differentiation towards the tenogenic lineage and to preserve the biomechanical properties of tendons. Thus, with the purpose to engineer tendon tissue, others moved cell-reseeded decellularized tendon matrices out of rotating tubes into bioreactors to apply uniaxial stimuli.^1^,^17^,^26^ The use of bioreactors for this purpose still has some drawbacks, such as the frequent handling of the constructs and high risks of culture contamination due to open or shared chambers. Notably, the culture chambers of these bioreactors do not permit a simultaneous treatment of several samples under different culture conditions and mechanical stimulations. More recently, multi-chamber bioreactors have been developed to independently control the aforementioned parameters. Qin *et al*.^14^ customized a stimulation device that accommodated four constructs in each chamber with its own regulation of the culture condition, except for cyclic strains. Others developed an enclosed modular vessel that combined a single construct culture with an individual mechanical stimulation.^30^ However, both these studies did not provide perfusion of the culture media that is important to maintain the homeostasis in 3D engineered constructs.^4^,^19^,^22^

To functionalize cell-seeded decellularized tendons, we developed a custom-made bioreactor consisting of multiple, independent culture chambers able to combine a bidirectional oscillatory perfusion with an intermittent, uniaxial strain and force feedback. Based on a previously validated oscillating perfusion bioreactor (OPB),^10^,^19^,^22^ our new system lodges tendon constructs and independently controls the loading regime of each tendon for strength and deformation during the course of the experiments. This automated bioreactor is cost-effective and user-friendly thanks to the absence of complex circuitry and of a perfusion pumping system. To assess the bioreactor functionality, our system was validated *in vitro* by culturing cell-reseeded decellularized tendon matrices.

## Materials and Methods

### Oscillating Stretch-Perfusion Bioreactor (OSPB) Design and Prototyping

Based on an already validated oscillating perfusion bioreactor (OPB) platform successfully used to generate engineered cartilage, bone and myocardial constructs,^4^,^10^,^19^,^22^ the new culture system—called oscillating stretch-perfusion bioreactor (OSPB)—was designed to apply a pump free, non-confined perfusion and a mechanical stretch to the cultured tendon constructs. The device was developed following main requirements: (i) independent chambers that fit on an oscillating platform exploiting perfusion and medium oxygenation working principles; (ii) sterilizable, biocompatible and chemically inert chamber materials; (iii) easy removable culture chamber from the bioreactor to be handled under laminar flow hoods; (iv) chambers provide a tissue holder consisting of a clamping grip to safely block the tendon without damaging the tissue; (v) the holder accommodates samples with clinically relevant dimensions; (vi) each chamber provides an axial stretching to the constructs with a preload and physiological-like stress/strain that could be programmed, real-time controlled and monitored during culture through a feedback loop control; (vii) the entire bioreactor system must be accommodated into standard cell culture incubators.

The OSPB chamber was designed using 3D CAD (computer aided design) software (Solid Edge ST6, Siemens). Then, to verify the design of the culture chamber to achieve a uniform fluid flow on the surface of constructs, computational flow dynamics (CFD) analyses were performed with a commercial software (COMSOL Multiphysics 4.2a). The analysis was performed through the CFD Module. The model was composed by the fluidic domain representing the culture medium, set as a homogeneous, incompressible Newtonian fluid with 1000 kg/m^3^ density and 8.1 × 10^−4^ Pa·s viscosity. A tetrahedral mesh with 1,500,000 elements from 3.24 × 10^−4^ mm to 0.001 mm was used. The Navier–Stokes, the Brinkman and the mass continuity equations for incompressible flow fluids were solved. To study the velocity profile and flux streamlines, a normal inflow velocity of 500 *µ*m/s was set at the inlet surface and a null pressure at the output.

**Figure 1:**
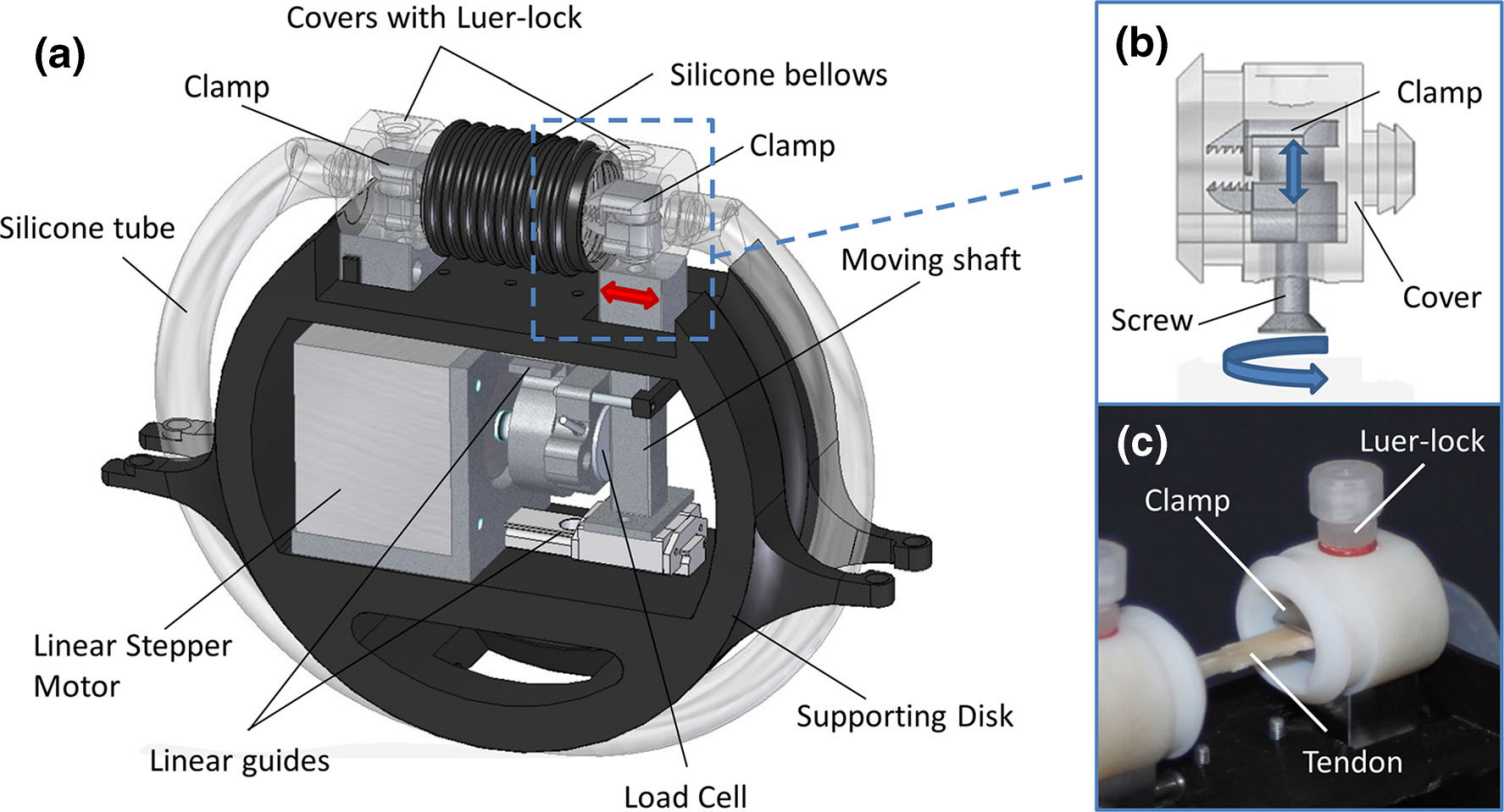
OSPB chamber. (**a**) CAD rendering of the new OSPB chamber; (**b**) detail of the clamp system and cover; (**c**) picture of a decellularized tendon fixed in the chamber.

A schematic depiction of an OSPB chamber is reported in Fig. 1. Each culture chamber was composed of two tissue holders, a platinum cured silicon tube (TYGON^®^ 3350, Cole-Parmer), a silicone bellow and three Luer-lock connectors for filling and changing the medium, all connected in a closed loop. Each chamber was lodged on a 3D printed supporting disk fitting in the OPB platform. The supporting disk also contained all the mechanical parts to activate the stretching phase during the dynamic culture (Fig. 1a).

**Figure 2:**
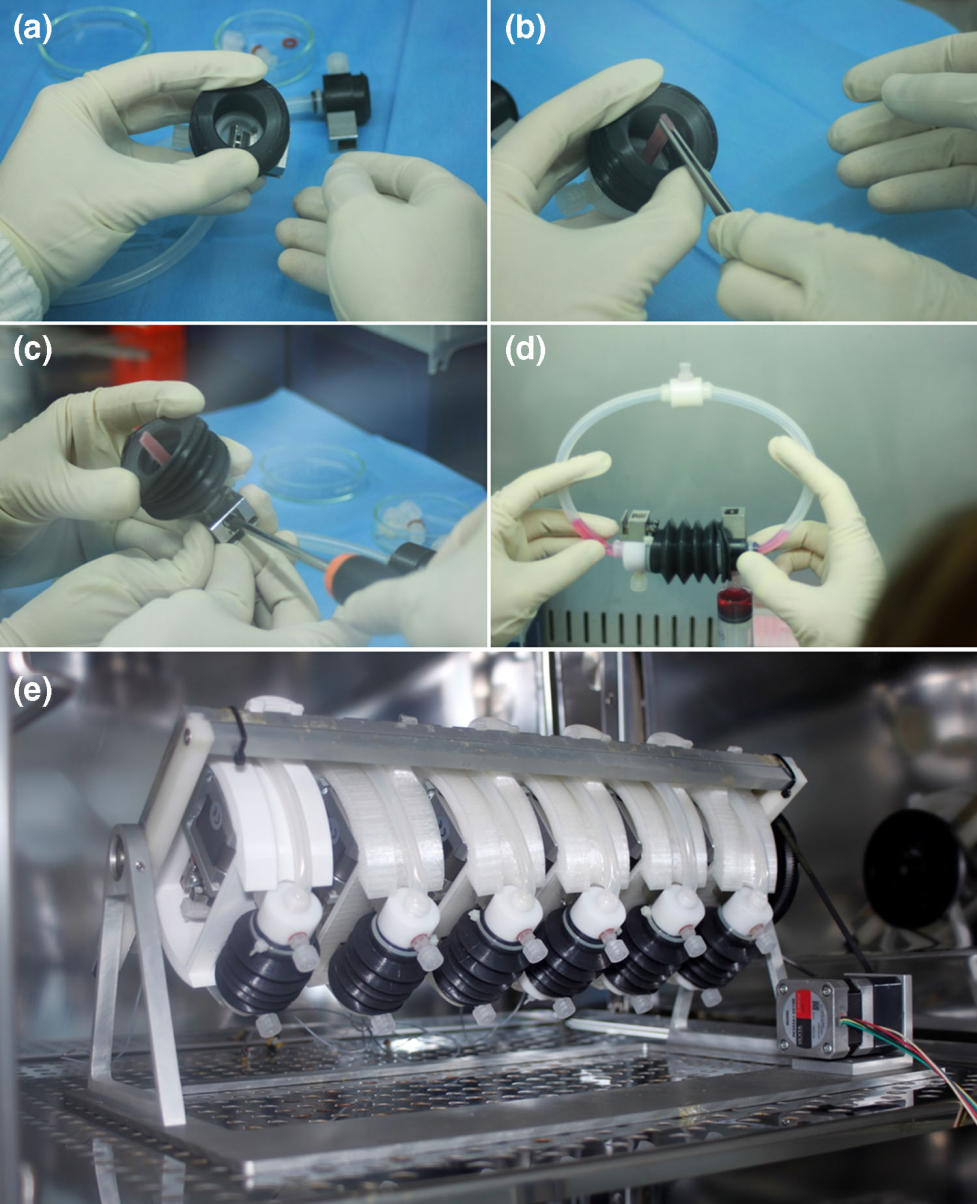
Assembly of the OSPB chamber and final system. (**a**); (**b**) the construct was firmly fixed in the clamp using tweezers; (**c**) the two clamps located on both sides of the chamber were activated thanks to an external screw with a screwdriver; (**d**) the chamber was closed and filled with culture medium; (**e**) complete OSPB system consisting in six separate chambers mounted on the OPB oscillating platform and placed in a standard cell culture incubator.

In particular, each tissue holder consisted of two main parts: a clamp made of stainless steel AISI 316L and the clamp cover made of Delrin^®^, as biocompatible materials for cell culture, all custom made and manufactured by high-precision machining (G3 snc). Indeed, to firmly lock the tendon constructs within the tissue holders during the stimulation and culture phases, a clamp system was developed to be accessible through an external management to maintain the sterility (Figs. 1b and 1c). In fact, the clamp covers provided a lower hole to operate on the clamp system and an upper threaded hole for lodging a Luer-lock for the culture medium. To secure the grip, the clamp system was designed with two alternate pattern rows of teeth which fit together when closed (Fig. 1b). The supporting disks were obtained through fusion deposition modeling with a 3D printer (BFB3000, 3D System). Each supporting disk was developed to allocate a stepper linear actuator, two miniaturized high precision linear guides (Schneeberger S.r.l. Angera), a load cell (BC302 60 kg, S2Tech), and two supports to fix the culture chambers. The stretching was generated by a linear actuator and transmitted to the cultured tendon construct through a moving shaft. The cell load with a capacity of 600 N was located between the linear actuator and the shaft for the real-time measurement of the force (N). Moreover, up to six parallel supporting disks can be magnetically allocated and removed independently in the OPB platform. The OSPB chamber allowed performing non-confined perfusion and stretching at the same time in an automatic way, without user manipulations. As aforementioned, the chamber was composed of a sterile part that contained both the construct and the culture medium, and a supporting part containing the mechanical components to generate the stretching stimulation. The assembly of the chamber took place under a laminar flow hood to ensure the sterility, as shown in Fig. 2.

### Development of the OSPB Control System

A PC-based control system was developed by using the LabVIEW development environment (National Instruments). A multipurpose DAQ-MX I/O board (NI USB-6211) and an Arduino Mega were used for linking the stepper motor controllers and the load cell amplifiers with the PC. The control system allowed to individually control each chamber for frequency (0.1–3 Hz), strain (up to 7%) and timing of activation. The flow speed was controlled by the OPB controller. The control system was programmed to alternate a cyclic strain and perfusion phase with a rest phase during which only the perfusion was active. Thanks to a feedback loop control, the constructs were individually preloaded to assure the proper tension before every strain and perfusion phase.

### Validation Testing of the OSPB

To test the functionality of the OSPB, a decellularized tendon was fixed in the chamber. The stimulation patterns were set through the user interface of the software, where frequency, deformation and timing of the stimulation can be set for each chamber, independently.

To evaluate the performance of the construct holder grip and the system stability, a pre-programmed deformation of 1, 3, 5 and 7% at 0.33 Hz of frequency was applied progressively. The preload was set at 3 N.

### *In Vitro* Validation of the OSPB

#### Scaffold Preparation and Cell Seeding Procedures

Equine superficial digital flexor tendons (SDFT) were chosen because of xenografts are becoming even more used for reconstructive surgery.^8^ SDFT specimens were harvested from the forelimbs of three adult horses (geldings, mean age 10 ± 5 years) at the slaughterhouse. SDFT specimens were decellularized and terminally sterilized through *β*-irradiation based on our previous studies.^2^,^12^ Briefly, the decellularization of SDFTs was performed under agitation in 1% tri-*n*-butyl-phosphate buffered in 1MTris-HCl for 24 h at room temperature, then rinsed in distilled water and stored in phosphate buffer at 4 °C to remove detergents. After treating in 0.0025% DNAse-I, SDFTs were incubated in 3% peracetic acid solution under agitation at room temperature for 4 h, then rinsed in phosphate buffer. Finally, samples were *β*-irradiated at 15 kGy and frozen dry at −80 °C until use.

To reseed decellularized tendon matrices (DTMs), rabbit bone marrow mesenchymal stem cells (rbBMSCs, Oricell™, Cyagen Biosciences, Inc.; Cat. No. RBXMX-01001, passage 2) were chosen according to our expertise on their behavior under specific stimuli *in vitro*.^3^,^6^ The rbBMSCs have been widely studied and chosen by the authors in order to conduct future studies in predictable animal model of tendon reconstruction, such as testing the efficacy of the engineered tendon constructs in a rabbit model of Achilles tendon full transection. The rbBMSCs were expanded at a density of 6000 cells/cm^2^ in culture medium consisting in high glucose Dulbecco’s modified Eagle’s medium containing 4.5 g/L glucose (Gibco), 10% fetal bovine serum (Hyclone), 100 U/ml penicillin–streptomycin, 2 mM l-glutamine, 1% sodium pyruvate, 1% HEPES (all from Gibco), and 10 ng/ml basic fibroblast growth factor (Peprotech).^6^ Fresh medium was changed twice a week and cells were used after reaching 90% of confluence. Before cell seeding, frozen DTMs were shaped in slices of 43 mm length, 5 mm width and 3 mm thickness with a precision saw (IsoMet™ 4000, Buehler) to reduce any manual variability. Then, twelve injections of 20.000 rbBMSCs/20 µl were performed perpendicular to the DTM fibers throughout their length with a 30G needle. The DTM surfaces were also seeded with 50.000 rbBMSCs/cm.^2^ To promote cell adhesion, all constructs were statically cultured in Petri dishes at 37 °C, 5% CO_2_ for 48 h. Then, six independent samples underwent dynamic culture (DC) in the bioreactor for 7 days, and six independent samples were also cultured in static conditions for the following 7 days (SC), as controls. For each condition (DC and SC), three constructs derived from three different tendon donors were processed coupled within the same experimental setup. The experiments were then repeated in duplicate.

#### Construct Culture in Dynamic Conditions

After 48 h, six replicates were transferred into the OPSB system to carry out the dynamic culture (DC). Specifically, under sterile conditions, constructs were fixed at their edges by means of tissue holders, then, the six independent culture chambers containing one sample each were closed with chamber covers, and the culture medium (27 ml/chamber) was injected through the Luer-lock entries to perfuse the constructs at 100 *µ*m/s.

The OPBS system controlled the frequency (0.33 Hz) and amplitude of the strain (3%) applied to the constructs, as also suggested by others.^14^ Three running cycles of 15–30–60 min of activity alternated with 15–30–60 min off were repeated two times per day, followed by a rest phase for 7 days to gradually adapt seeded cells and constructs to mechanical stimulation over time. The culture medium was changed twice a week during the rest phase over the course of the experiment.

After 7 days of culture, SC and DC samples were analyzed for cell viability and distribution, cell morphology within and onto the DTMs, type I and III collagen deposition, and DTM surface ultrastructure.

#### Cell Viability Assay

To evaluate rbBMSCs viability and distribution onto the DTM surface, Live & Dead assay (Life Technologies) was performed according to the manufacturer’s instructions. Briefly, both SC and DC constructs were labelled with Live & Dead stain consisting in 2.5 *μ*l calcein AM and 10 *μ*l ethidium homodimer-1 dissolved in 5 ml of phosphate buffer saline. Then, samples were incubated at 37 °C in the dark for 15 min and microscopically analyzed at ×2 magnification (Olympus IX71 fluorescent microscope).

#### Qualitative and Quantitative Histology and Immunohistochemistry

SC and DC constructs were fixed in 10% buffered formaldehyde for 24 h, dehydrated, paraffin embedded, and sectioned at 3.5 *μ*m, then stained with Haematoxylin–Eosin staining (H&E) to evaluate the morphology of cells and of the newly formed ECM within the constructs.

To evaluate type I and III collagen, immunostaining was performed with primary anti-collagen type I (1:200 dilution) and type III (1:2000 dilution) for 60 min. Specifically, the following antibodies were supplied from Sigma-Aldrich: monoclonal anti-collagen type I (mouse IgG1 isotype; C 2456) and monoclonal anti-collagen type III (mouse IgG1 isotype; C 7805).

Then, sections were exposed to a biotinylated anti-mouse IgG secondary antibody (1:200 dilution; Vector Labs BA-2000) for 30 min. The signal was detected by the streptavidin–biotin method coupled with the 3′-Diaminobenzidine (DAB) chromogen system (Vinci Biochem). Sections were then counterstained with haematoxylin. The negative control was carried out by omitting the primary antibodies. Photomicrographs of different regions within each construct were captured using Olympus IX71 light microscope, a ×20 objective and Olympus XC10 camera.

To quantify the coherency and the local alignment of the new collagen deposition within the inner injected channels, ImageJ plug-in OrientationJ (Version 19.11.2012) was employed. Specifically, on each SC (*n* = 6) and DC (*n* = 12) microphotograph, three independent regions of interest (260 × 200 px) within the inner channels were selected, thus collagen fiber orientation and coherency were measured to discriminate between significantly and not-significantly oriented areas (1, highly oriented structures; 0, isotropic areas). Coherency data were statistically analyzed through Graph Pad Prism 5 software (Graph Pad Software, Inc, La Jolla, USA). Shapiro–Wilk test was used to assess the normal distribution of data. Data obtained for normal distributed values were analyzed using an unpaired test and reported as mean ± SEM.

#### Scanning Electron Microscopy

To study the surface of the reseeded and unseeded DTM constructs, SEM analysis was carried out. Specifically, SC, DC and DTM samples were fixed in 2.5% paraformaldehyde and 2.5% glutaraldehyde in 0.1 M Na-Cacodylate buffer (pH 7.4). After fixation, the samples were rinsed with Na-Cacodylate buffer and fixed for 1 h in OsO4 (1% in Na- Cacodylate buffer), then dehydrated by ascending scale of ethanol (50, 70, 90, 100%) for 20 min each, mounted on aluminium stubs, and sputter-coated with gold using a SEMPREP 2 Sputter Coater (Nanotech Ltd). Observations were performed with a LEO 1400 EVO Scanning Electron Microscope (Zeiss) mixing secondary and backscattered electrons detectors. Images from different regions within each construct were acquired at 20 kV at a working distance of 11–30 mm.

## Results

### CFD Velocity Profile

The internal geometry of the scaffold holder was optimized to achieve a uniform non-confined perfusion within the tendon construct. To this aim, CFD analyses were performed by modeling the culture medium domain. Fluid dynamic analyses were performed considering half portion of the scaffold holder with the culture medium flowing from the tube to the chamber and evaluating the velocity profile near the construct. CFD studies showed a uniform perfusion speed of 100 *µ*m/s on the construct surface. Peaks of velocity above 800 *µ*m/s were revealed near the clamp with a rapid reduction of the velocity once entering the chamber. The streamlines in the chamber had not revealed turbulences (Supplementary_Figure 1S).

### Performance and Operation of the OSPB

All chambers were assembled under a laminar flow hood to ensure the sterility and then, up to six chambers at the same time were mounted on the OPB oscillating platform. The entire bioreactor system was placed in a standard cell culture incubator allowing long-term culture in a controlled environment (Fig. 2e). The chambers were individually connected to the control system through two connectors per chamber, one to control the stepper linear motor and one for the load cell signal.

The user interface of the software showed in real-time the axial force applied by the linear actuator to the construct thanks to a load cell. To preload the tendon construct, the closed-loop control system allowed increasing gradually the stroke of the piston up to the desired load using the PID (proportional, integrative, derivative) controller. Once the preload was achieved, the software automatically generated the stimulation patterns for each culture chamber. A visual alarm alerted in the case of preload or grip failures. The developed control system was able to preload the tendon manually or automatically setting a fixed loading value.

**Figure 3:**
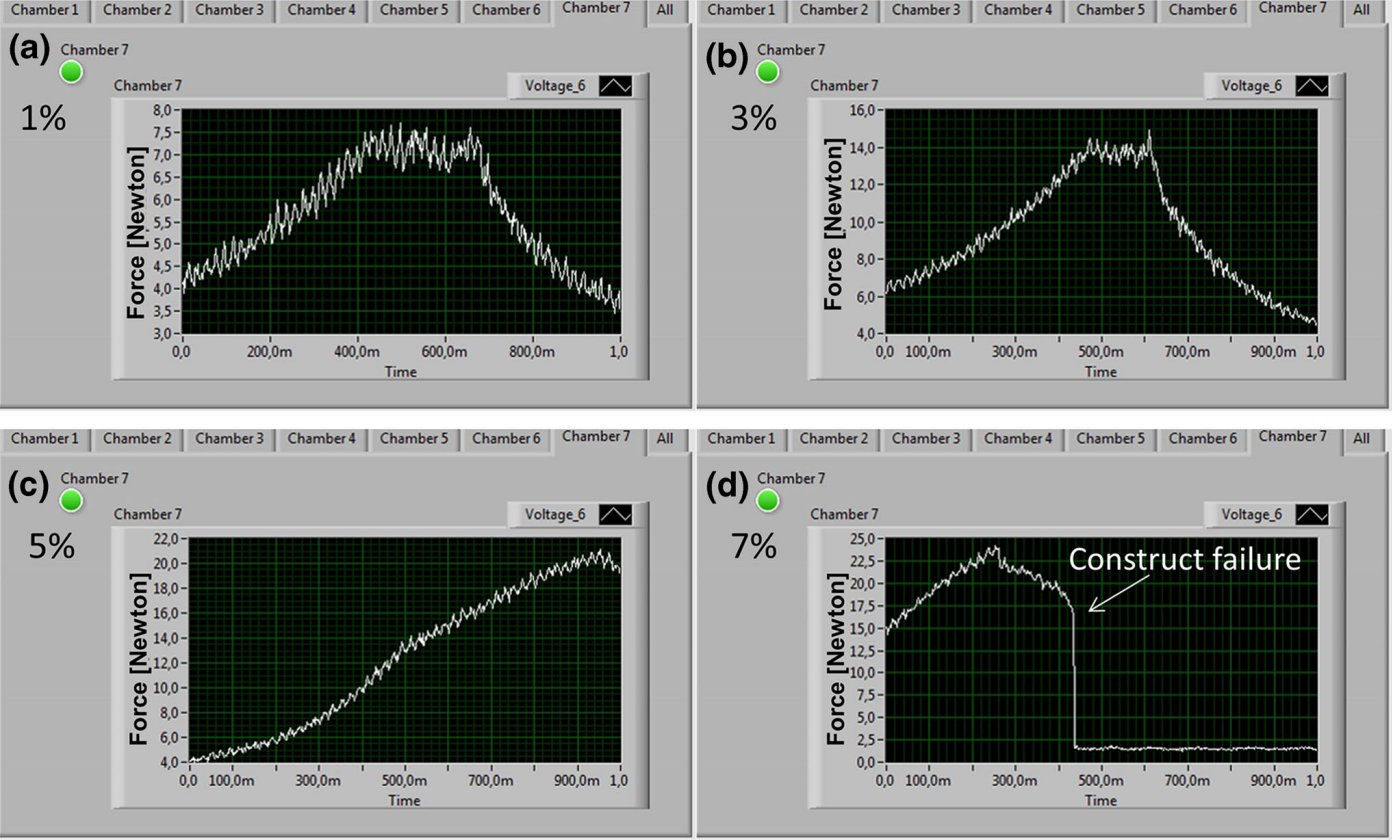
A detail of the user interface of the OSPB software. (**a**); (**b**); (**c**) Stretching force measured by the load cell mounted in the chamber stimulates at different strains (1, 3, and 5%); (**d**) Software detection of unexpected construct failures. *Y* axes reported force in Newton.

In addition, the user interface allowed setting the culture parameters (strain, frequency and cycle timing) of each chamber in a precise and reproducible way. For instance, during the preliminary mechanical tests, an increasing stretch pattern, from 1 to 7% at a frequency of 0.33 Hz, was imposed on the decellularized tendon. Figure 3 shows the trend of the axial force measured by the load cell. At 7% of deformation (Fig. 3d), a failure of the tendon constructs was simulated, demonstrating the ability of the system to highlight unexpected events by capturing through the feedback system and software and graphically showing it on the monitor.

After 7 days of dynamic culture, the recording of the load cell demonstrated that the entire preload, stretching and rest processes were correctly performed daily during the experimental time, without any additional intervention by the operators except for the medium change.

### Cell Viability Assay

**Figure 4:**
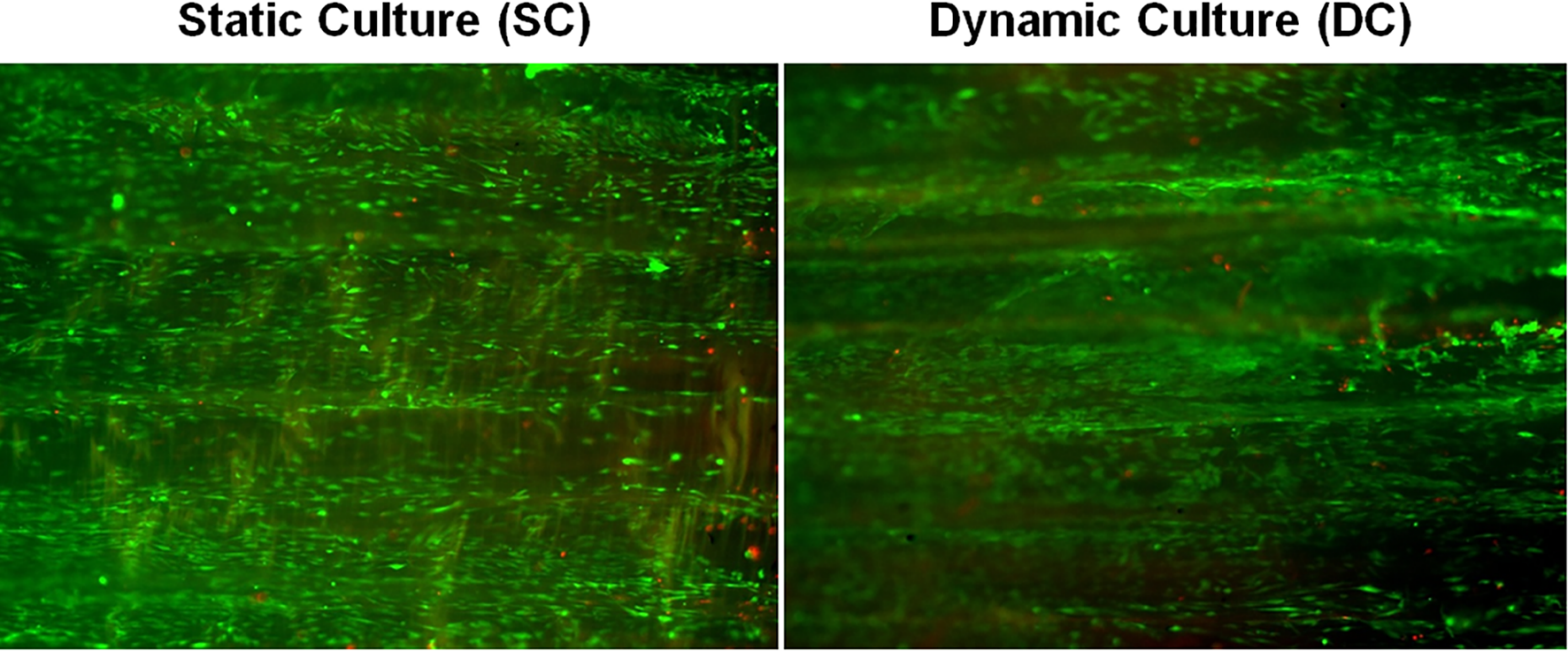
Live and Dead staining. Cell viability and distribution is reported for the static and dynamic culture after 7 days of stimulation.

Live & Dead Viability shown in Fig. 4 demonstrated that in both groups, rbBMSCs were mostly viable and homogeneously distributed onto the entire matrix surface after 7 days of culture. Qualitatively, the total number of cells was similar between SC and DC, and the number of dead cells was only slightly increased in the DC group. More importantly, cells distributed on the surface of the matrix appeared more round-shaped and connected by a thin, loose matrix in the SC compared to the DC group, in which cells appeared elongated.

### Qualitative and Quantitative Data from Histology and Immunohistochemistry

**Figure 5:**
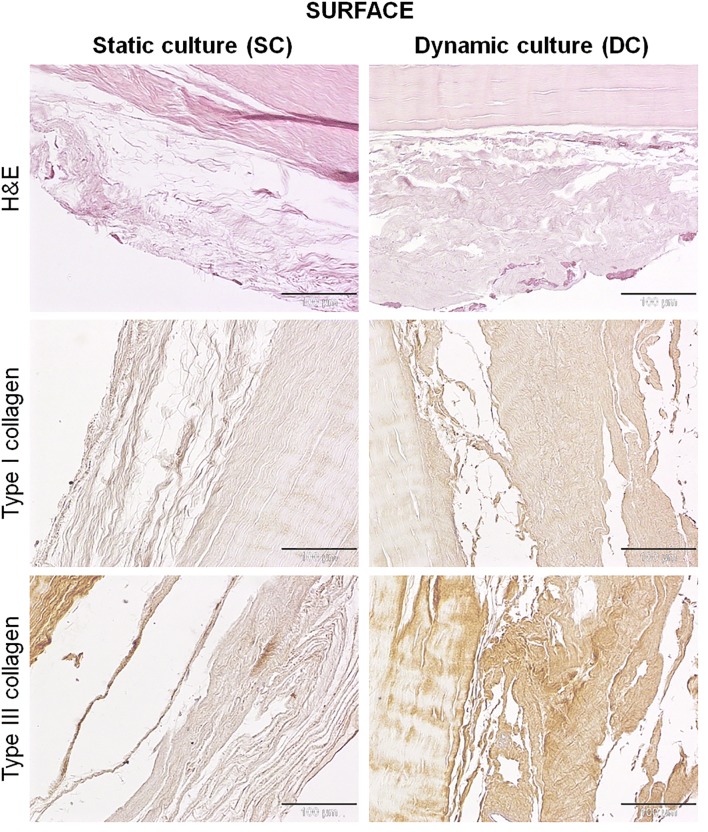
H&E and collagen type I and III immunostaining of samples, 7 days after static or dynamic culture. On the surfaces of constructs, the newly synthesized extracellular matrix appeared thinner, looser and less organized in the SC constructs, whereas in the DC group it was more dense and structured. The newly synthesized extracellular matrix is generally poor in type I collagen, but rich in type III collagen. DC constructs produced a more positive type I and type III collagen than that deposited by the SC group. Magnification ×200, scale bars 100 µm.

No differences were found between SC and DC cultured constructs regarding the structure and organization of the collagen fiber bundles within the tendon matrix. On the surfaces of both SC and DC constructs, a layer of newly synthesized ECM was detectable (Fig. 5). This matrix appeared thinner, looser and less organized in the SC constructs, whereas in the DC group it was more dense and structured. In both groups, cells appeared mostly rounded, especially in the SC group, and some of them were on the surface of the newly synthesized matrix, while others penetrated in it in the DC group.

The immunostaining showed that this newly synthesized ECM was generally poor in type I collagen, but rich in type III collagen (Fig. 5). In particular, ECM synthesized by rbBMSCs cultured in DC appeared qualitatively more positive for both type I and, to a lesser extent, type III collagen than that deposited by the SC group.

**Figure 6:**
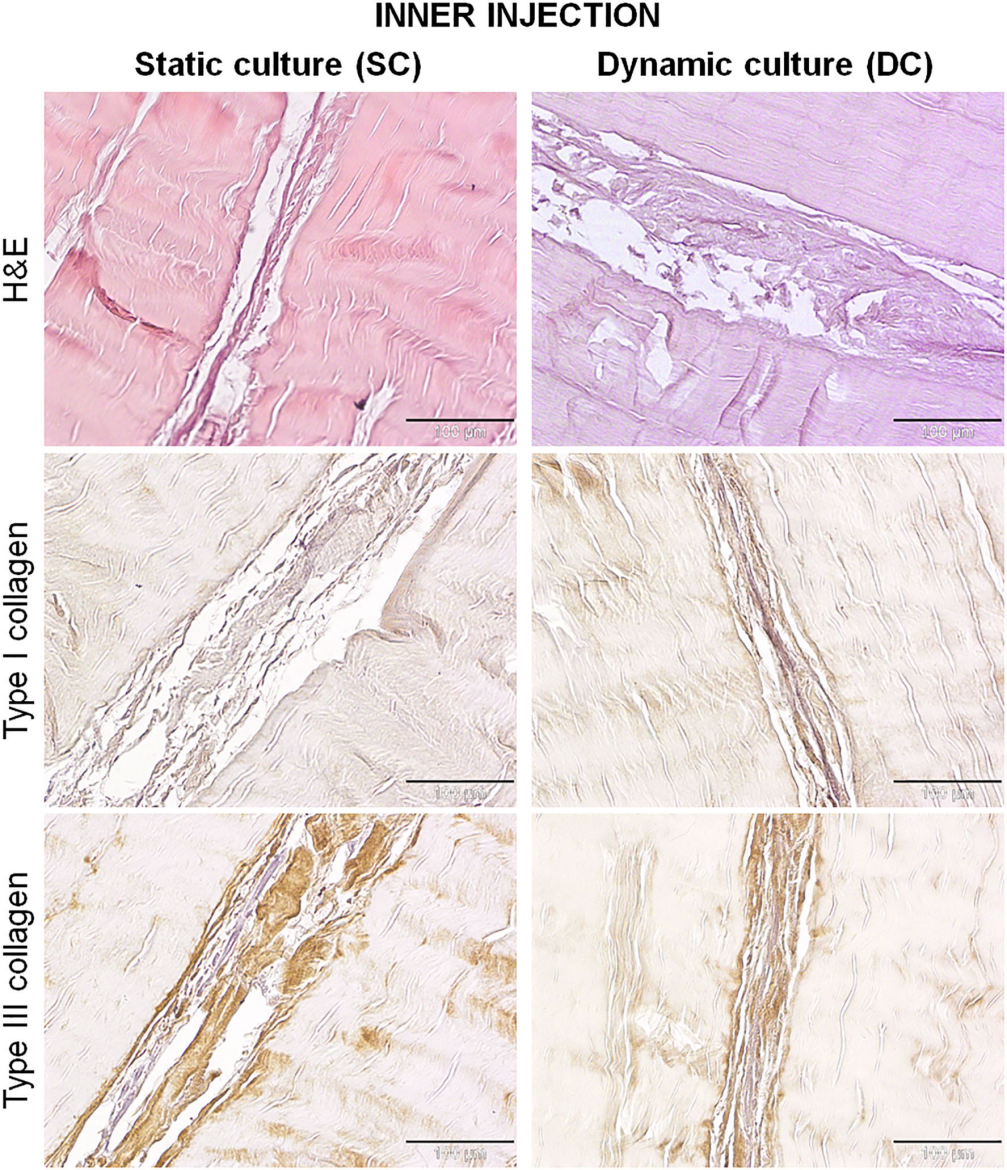
H&E and collagen type I and III immunostaining of samples, 7 days after static or dynamic culture. Within the cell injected fibers, the newly synthesized extracellular matrix appeared thinner, looser and less organized in the SC constructs, whereas in the DC group it was more dense and structured. The matrix deposited by DC was qualitatively little more positive for type I collagen than that synthesized by SC, while type III collagen was comparable between the two groups. Magnification ×200, scale bars 100 *µ*m.

Regarding the rbBMSCs injected within the DTMs, in both groups, cells did not repopulate homogeneously the tendon matrix, but they were found within channels created between adjacent fiber bundles along the whole length of the constructs, as shown by H&E staining (Fig. 6). These channels were wider in the DC constructs and, similarly to what occurred on the surface, the cells were able to synthesize an ECM. Specifically, rbBMSCs cultured in DC produced a greater amount of matrix that also appeared more dense and structured compared to that synthesized by the cells cultured in SC. In both groups, the inner injected cells assumed a more tenogenic-like morphology than those cultured onto the surface, showing a more elongated and thin shape with a scarce cytoplasm and small nuclei.

As seen on the construct surface, the newly synthesized matrix in the inner injection resulted richer in type III than type I collagen (Fig. 6). The matrix deposited by the DC group resulted qualitatively little more positive for type I collagen and poorer of type III collagen than that synthesized by the SC group.

**Figure 7:**
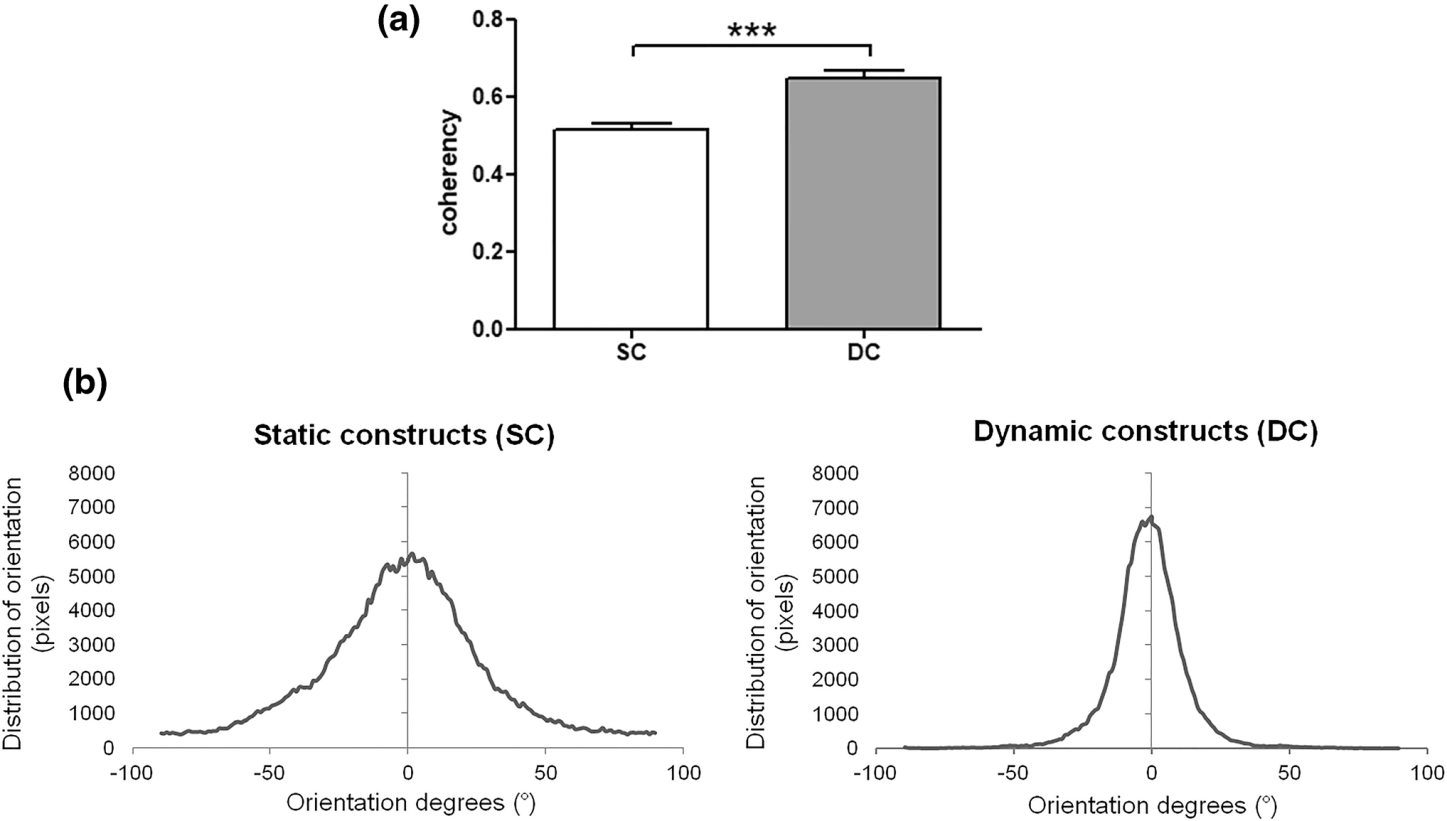
Quantitative data of the alignment and orientation of the newly formed fibers. (**a**) coherency between significantly and not-significantly oriented areas, *p *< 0.001***; (**b**) orientation histogram of the newly formed collagen fiber within the inner channels in SC and DC samples.

In Fig. 7a, the histogram reports the coherency between significantly and not-significantly oriented areas of the newly formed collagen matrix within the inner injected channels. The DC group showed a significant higher orientation compared to the SC group (*p* < 0.05). In Fig. 7b, orientation values were weighted by the coherency values to quantify the orientations that corresponded to the elongated fibers. A sharp peak around 0° was observed in the DC group histogram rather than in the SC samples, indicating a more elongated, parallel distribution of the new collagen fibers in the DC group.

### Scanning Electron Microscopy

**Figure 8:**
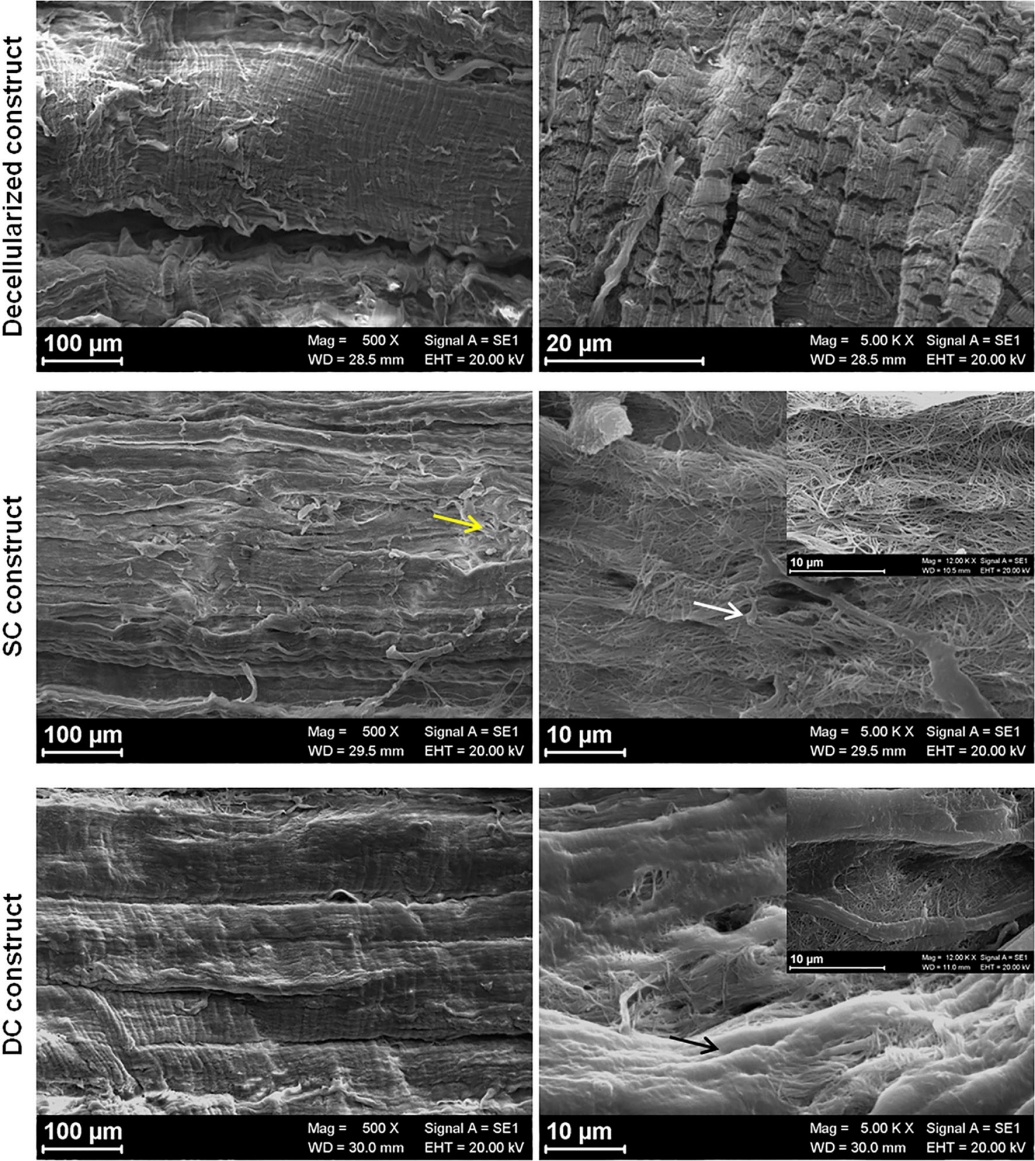
SEM imaging. At the lowest magnification (100 *µ*m), decellularized and SC tendons showed the classical tendon morphology with orderly, parallel collagen fiber bundles, even if some of them emerged from the matrix not following the general organization (yellow arrow). At higher magnification (20 *µ*m), cells were clearly visible on the surface of the fibrillar matrix of the SC constructs with protrusions departing from rounded cell bodies (white arrow). Whereas, in the DC constructs, cells appeared completely embedded within the dense matrix and cells were not clearly recognizable (black arrow).

Scanning electron microscopy (SEM) analyzed the ultrastructure of the reseeded constructs. SEM images did not show any sign of disruption or modification of collagen bundles alignment at the micrometer level in none of the samples. At the lowest magnification, the decellularized and SC tendon showed the classical tendon morphology with orderly, parallel collagen fiber bundles, even if some of them emerged from the matrix not following the general organization. On the contrary, after the dynamic culture, the surface of DC constructs appeared more organized and uniform and all the superficial collagen fibers followed the same orientation (Fig. 8).

At higher magnification, decellularized tendons showed the typical fiber pattern. For the reseeded constructs, it was possible to deeply analyze the matrix synthesized by rbBMSCs. In particular, the matrix deposited by cells cultured in SC appeared more fibrillary and the fibers of which it was composed were clearly visible and discernible, creating a sort of mesh covering the construct surface. Otherwise, cells were clearly visible on the surface of the fibrillar matrix with protrusions departing from rounded cell bodies. Conversely, the matrix formed by cells cultured in DC was denser and appeared as a unique structure; in fact, fibrils that constitute it were identified in specific areas of the surface. Furthermore, cells appeared immersed and covered by that dense matrix and were not clearly recognizable.

At higher magnification, the differences between the matrix synthesized onto the SC and DC constructs were more evident; in fact, the fibrillar nature of the matrix in the SC group was still clearly detected and the single fibril was still identifiable. On the contrary, the fibrils in the DC constructs were compacted and closely bound to each other and there were much smaller empty spaces in-between them.

## Discussion

The purpose of this study was the development of a bioreactor to dynamically culture biological constructs for the replacement of tendon defects. Through this TE advanced approach, it could be possible to produce functional engineered tendon substitutes to be used in clinics, in the next future.

Static cultures of engineered constructs are limited in nutrient supplies, waste removal and lacked of all physical stimuli necessary for a homogenous cell repopulation, and orientation as well as tissue-specific differentiation, and ECM production. Thus, dynamic and more complex culture conditions are required, especially in the case of highly oriented, dense collagen tissues like tendons. Among several dynamic systems developed to overcome these limitations, some authors designed bioreactors to perform mechanical stimulation or dynamic perfusion culture for tendon-based constructs. As demonstrated, the use of unconfined perfusion (rotating culture) improved the cell metabolism.^7^,^15^,^27^,^30^ Although rotating cultures supported the cellular nutrition, these systems could not ameliorate the cell distribution along the axis of the construct fibers because of the inability to orient the culture medium flow in a single direction. The use of fluid pumps and hydraulic circuitry can be useful to generate a directional flow, but implies a more complex and expensive approach.^19^ Conversely, one of the main advantages of our bioreactor system was to create a bidirectional flow without an external pump. To better distribute seeded cells onto scaffolds, others described the application of mechanical stimuli in the absence of a fluid perfusion.^1^,^17^,^18^,^26^ Moreover, a mechanical stimulation mimicking physiological loads improves the cellular production of collagen, and fiber orientation while preserving biomechanical properties of the tendon-derived scaffolds.^25^ However, to the best of our knowledge, none of the custom-made or commercial bioreactors allows a simultaneous pump-free fluid perfusion and an individual, custom mechanical stimulation. On the other hand, the developed OSPB allowed applying uniaxial cyclic tensile stimulation along with the culture medium unconfined perfusion, thus increasing the nutrient supply for cell metabolism and the elongated distribution of cells onto the construct surface. More importantly, the flow velocity verified through CFD produced shear forces that did not negatively compromise the cell viability and phenotype when seeded onto the construct surface.^20^

Another advantage of the OSPB was the possibility to independently stimulate each chamber containing a single construct, differently from other devices that can lodge independent samples, but apply the same stimulation patterns simultaneously.^1^,^14^,^17^,^18^ According to this approach, the OSPB advantaged both the experimental *in vitro* studies and the potential clinical translatability. Indeed, OSPB operates on every single construct and employs different stimulation protocols and harvesting/control time points independently, within the same experiment. Another important improvement of the OSPB system is the automation through specifically designed, user-friendly software that includes a feedback control. Conversely, some authors developed a bioreactor for tendons able to generate an automated cyclic stimulation by applying a manual pre-load, but without a direct feedback or the possibility to modify the strain during the experiments.^16^ On the other hand, the OSPB system allows to automatically reach the desired pre-load using a closed-loop feedback control. Moreover, the OSPB software can be pre-programmed to manage and change automatically the stimulation patterns of each chamber during the course and over the time of the experiment. Finally, the entire OSPB system can be lodged in a standard cell culture incubator and handled under the laminar flow hood, guaranteeing sterile conditions. CFD analysis verified the absence of turbulence and the flow speed on the surface of the construct during the perfusion phase. Differently from our previous work,^23^ in which the simulation of the development of the fluid flow was deeply assessed, in this case, the simulation was simplified avoiding the analysis of the fluid during the cyclic stretching phase. Indeed, the simulation was computationally complex because of the simultaneous effects of both the bioreactor oscillation and chamber stretching. Because of the distinctiveness of our customized bioreactor, the heterogeneity of studies in terms of the types of seeded cells, dynamic culture devices and protocols, and scaffold materials,^8^ the results obtained by the *in vitro* validation of the OSPB are not directly comparable with other studies in the current literature.

Although contrasting opinions support or not the benefit of cell injection within the construct fibers,^17^,^21^ we associated an outer and an inner cell colonization to evaluate the effects of the dynamic culture on exposed or inter-fiber injected cells and to guarantee a high cell engraftment within the engineered constructs. Indeed, it has been strongly demonstrated that, in the presence of a dynamic stimulation, only a 10–20% of cells superficially seeded remains on the surface of the engineered scaffold.^1^,^17^ In our series, the cell viability was equally maintained both in the SC and DC groups. However, in the DC group, the seeded cells appeared more elongated along the axis of the collagen fibers, as also demonstrated elsewhere.^24^ Moreover, cells were deeply embedded in the newly synthesized matrix on the surface of the DC construct compared to SC, as clearly visible at SEM. The most encouraging results are represented by the type of newly formed ECM, both on the surface and within the inner channels in the DC group, in which it appeared denser, organized and richer in type I and III collagen compared to SC group. The presence of type III collagen is mainly involved in the early stages of tendon healing. This finding is consistent with that described by others who showed that cell-seeded constructs increased the collagen production in response to loading forces.^17^,^28^ However, both these studies did not quantify or verify the presence of type I or III collagen produced by the seeded cells. The promising production of well-organized, well-oriented newly formed ECM fibers of DC group, as quantitatively demonstrated in the present study, could be related to the application of gradual running cycles of 15–30–60 min on–off repeated two times per day that permitted the adaptation of the seeded cells and the construct to the mechanical stimulation over time.

Despite the present study lacks in direct biomechanical testing, the *in vitro* validation showed a more organized ECM synthesized by rbBMSCs loaded onto and within the decellularized tendon fibers in samples undergoing dynamic culture in the OSPB. Indeed, compared with static culture, it has been widely demonstrated that cyclic load can guide the cell alignment along the axis of the collagen fibers with resulting superior biomechanical properties, mainly ultimate tensile strength and elastic modulus, compared to static conditions.^1^,^17^ Similarly, we have already demonstrated that decellularized tendons maintained the biomechanical properties of the native tissue.^2^,^12^

Although the presence of type I and III collagen in our series presumes the up-regulation of collagen genes, future studies are required to investigate *in vitro* the gene expression of tenogenic markers and the cell differentiation in the conditions presented in this study.

In conclusion, our findings demonstrated that cell-seeded decellularized tendon grafts undergoing cyclic load in our bioreactor had a superior production and organization of newly ECM compared to static cultured constructs. This interesting result needs to be evaluated in animal models to investigate the *in vivo* response to the engineered tendon constructs after transplantation for a translational application to orthopedics. Above all, the designed OSPB could be considered a unique, cost-effective system able to support multiple independently controlled chambers in terms of biological and mechanical protocols, and allowing for parallel processing of several customized tendon constructs. This device could be employed in GMP and clinical settings thanks to the capability process independently and keep isolated each construct, thus preventing possible cross-contaminations and allowing several customized implants for different patients.

## Electronic supplementary material

Below is the link to the electronic supplementary material.

Supplementary material 1 (DOCX 178 kb)

## Abbreviations

CAD: Computer aided design
CFD: Computational flow dynamics
DC: Dynamic culture
DTMs: Decellularized tendon matrices
ECM: Extracellular matrix
FTE: Functional tissue engineering
OPB: Oscillating perfusion bioreactor
OSPB: Oscillating stretch-perfusion bioreactor
rbBMSCs: Rabbit bone marrow mesenchymal stem cells
SC: Static culture
SDFT: Equine superficial digital flexor tendons
