# Supplementary material for: Independent, Controllable Stretch-Perfusion Bioreactor Chambers to Functionalize Cell-Seeded Decellularized Tendons
Source: Ann Biomed Eng. 2019 Apr 8;48(3):1112–26. doi: 10.1007/s10439-019-02257-6 (PMC7015956; doi:10.1007/s10439-019-02257-6)
Supplement: Supplementary file 1 — Supplementary material 1 (DOCX 178 kb) [file 10439_2019_2257_MOESM1_ESM.docx]

Supplementary_Figure 1S. Fluid dynamic CFD computational analyses. (a) model representing the medium and the tendon construct domains; (b) colorimetric maps of the flux streamlines showing laminar flow and no turbulences; (c) colorimetric maps representing fluid flow velocity in the culture chamber.
